# Patterns of Recurrence and Survival After Pelvic Treatment for Locally Advanced Penile Cancer

**DOI:** 10.1016/j.euros.2022.11.005

**Published:** 2022-12-15

**Authors:** Hielke M. de Vries, Sarah R. Ottenhof, Tynisha S. Rafael, Erik van Werkhoven, Floris J. Pos, Bas W.G. van Rhijn, Luc M.F. Moonen, Niels Graafland, Jeantine M. de Feijter, Eva E. Schaake, Simon Horenblas, Oscar R. Brouwer

**Affiliations:** a Department of Urology, The Netherlands Cancer Institute – Antoni van Leeuwenhoek Hospital, Amsterdam, The Netherlands; b Department of Biostatistics, The Netherlands Cancer Institute – Antoni van Leeuwenhoek Hospital, Amsterdam, The Netherlands; c Department of Radiation therapy, The Netherlands Cancer Institute – Antoni van Leeuwenhoek Hospital, Amsterdam, The Netherlands; d Department of Internal Medicine, The Netherlands Cancer Institute – Antoni van Leeuwenhoek Hospital, Amsterdam, The Netherlands

**Keywords:** Penile neoplasms, Pelvis, Lymph node excision, Survival, Recurrence

## Abstract

**Background:**

Penile cancer (PeCa) is rare, and the survival of patients with advanced disease remains poor. A better understanding of where treatment fails could aid the development of new treatment strategies.

**Objective:**

To describe the disease course after pelvic lymph node (LN) treatment for PeCa.

**Design, setting, and participants:**

We retrospectively analysed 228 patients who underwent pelvic LN treatment with curative intent from 1969 to 2016. The main treatment modalities were neoadjuvant chemotherapy, chemoradiation, and pelvic LN dissection.

**Outcome measurements and statistical analysis:**

In the case of multiple recurrence locations, the most distant location was taken and recorded as follows: local (penis), regional (inguinal and pelvic LN), and distant (any other location). A competing risk analysis was used to calculate the time to recurrence per location, and a Kaplan-Meier analysis was used for overall survival (OS).

**Results and limitations:**

The median follow-up of the surviving patients was 79 mo. The reason for pelvic treatment was pelvic involvement on imaging (29%), two or more tumour-positive inguinal LNs (61%), or inguinal extranodal extension (52%). More than half of the patients (61%) developed a recurrence. The median recurrence-free survival was 11 mo. The distribution was local in 9%, regional in 27%, and distant in 64% of patients. The infield control rate of nonsystemically treated patients was 61% (113/184). From the start of pelvic treatment, the median OS was 17 mo (95% confidence interval 12–22). After regional or distant recurrence, all but one patient died of PeCa with median OS after a recurrence of 4.4 (regional) and 3.1 (distant) mo. This study is limited by its retrospective nature.

**Conclusions:**

The prognosis of PeCa patients treated on their pelvis who recur despite locoregional treatment is poor. The tendency for systemic spread emphasises the need for more effective systemic treatment strategies.

**Patient summary:**

In this report, we looked at the outcomes of penile cancer patients in an expert centre undergoing various treatments on their pelvis. We found that survival is poor after recurrence despite locoregional treatment. Therefore, better systemic treatments are necessary.

## Take Home Message

In penile cancer patients undergoing various treatments on their pelvis, there is a strong tendency for recurrence and systemic spread. Despite the treatments, the prognosis remains poor, emphasising the need for better systemic treatments.

## 1 Introduction

Penile cancer (PeCa) is thought to exhibit a predictable stepwise lymphatic metastatic pattern [1]. From the primary lesion, cancer first spreads to the inguinal lymph nodes (LNs; pN1–2), then to the pelvic LNs (pN3), and ultimately to the para-aortal LNs or distant sites (M1) [1]. Distant metastases are atypical without concurrent LN spread [2]. Therefore, LN metastases are the main predictor of survival in PeCa patients, emphasising the need for effective LN treatment [3], [4]. Over the last decade, pelvic LN metastasis treatment consisted of different treatment modalities and combinations [5]. Historically, pelvic treatment consisted of surgery only. Adjuvant radiation was added for patients at a high risk of regional recurrence in the pelvic area [6]. In current guidelines, neoadjuvant chemotherapy is recommended in patients presenting with pelvic metastases on imaging [7]. However, not all pelvic metastases are seen on imaging, and high-level evidence showing effective neoadjuvant chemotherapy is lacking [8]. Available evidence also shows a high level of toxicity [9], [10]. In the group of patients without radiological signs of pelvic LN involvement with two or more tumour-positive ipsilateral inguinal LN metastasis or the presence of extranodal extension (ENE), surgical treatment of the pelvis (so-called prophylactic pelvic treatment) is recommended [7], [11]. Despite current treatment strategies, the 5-yr cancer-specific survival (CSS) of patients with pN3 disease is as poor as 37% [12]. However, the recurrence pattern after treatment with curative intent is not precisely known. A step towards understanding the high failure rate after pelvic treatment to improve treatment strategies in the future could be to evaluate whether, when, and where cancer recurs. Therefore, the main objective of this study was to describe the disease course after pelvic LN treatment for PeCa. Secondary objectives are the recurrence pattern, prognosis after pelvic treatment, and prognosis after recurrence.

## 2 Patients and methods

In total, 228 PeCa patients who underwent treatment of the pelvic nodes with curative intent between 1969 and 2016 were evaluated retrospectively. Patients for whom pelvic treatment was part of their primary treatment and those for whom it was part of the treatment for a recurrence were both included. Hospital records older than 1969 could not be tracked reliably. The medical record review was finalised in 2018 to enable a substantial follow-up. Patient and follow-up data were recorded from our institutional PeCa database. The eighth tumour-node-metastasis (TNM) classification was used. Previously, tumours were restaged retrospectively by an expert uropathologist to the seventh TNM classification, from which they were updated to the eighth version using the previously revised pathology reports [12]. Clinical N stage was based on staging with palpation and ultrasound from 2001 prior to dynamic sentinel node biopsy or inguinal treatment. Pathological N stage was recorded as not evaluable (pNx) for patients treated with chemoradiation or neoadjuvant chemotherapy. Missing items were collected from the electronic patient records or digitalised written records. The institutional review board approved this study (IRBd20-001) and waived the need for informed consent.

Different treatment protocols for pelvic LNs have been applied over the years [12]. In short: in 1988, indications for prophylactic pelvic lymphadenectomy after inguinal dissection were formulated together with standardised follow-up and imaging. Patients with pelvic involvement on imaging underwent pelvic lymphadenectomy. In 2008, neoadjuvant chemotherapy was added to treat patients with radiologically (and mostly cytologically proven) evidence of pelvic nodal involvement. The rationale was based on an analysis of treatment results and worldwide trends [13], [14]. Patients with locally advanced primary tumours or irresectable inguinal metastases were also treated with neoadjuvant chemotherapy or chemoradiation [9]. From 2005, imaging changed gradually from computed tomography (CT) to fluorodeoxyglucose (FDG)-positron emission tomography (PET)/CT [15]. From 2013, neoadjuvant chemotherapy was gradually replaced by chemoradiation, followed by surgery only in case of residual disease [16]. Solely patients treated with curative intent were included in the current study.

Four main treatment categories were defined: prophylactic pelvic LN dissection (PLND), therapeutic PLND, neoadjuvant chemotherapy, and chemoradiation. A therapeutic PLND was performed in case of suspicious pelvic LNs on imaging. Our centre applied the following neoadjuvant chemotherapy regimens: methotrexate/bleomycin/cisplatin, cisplatin/5-fluorouracil (FU)/docetaxel (TPF), or cisplatin/5-FU. The indication for the various regimens was at the discretion of the medical oncologist. During response evaluation, residual disease determined by pelvic imaging was mostly treated with surgery. Chemoradiation consisted of 33 daily fractions of 1.5–1.8 Gray, with intravenous mitomycin on day 1 and capecitabine tablets on radiation days. Residual lesions after chemoradiation (on PET/CT) were resected surgically. Adjuvant radiation or chemotherapy after surgery was administered to patients with high-risk LN basins (multiple positive LNs or ENE). Pelvic imaging was based on CT or FDG-PET/CT, and performed prior to treatment in the presence of suspicious palpable inguinal LN or positive inguinal fine-needle aspiration cytology. Imaging was performed at least 6 wk after neoadjuvant chemotherapy or chemoradiation. Follow-up was calculated from the start of pelvic treatment until recurrence or death. We discussed all treatment decisions in our weekly uro-oncology multidisciplinary team meeting.

Recurrences after pelvic treatment were identified by physical examination, imaging, cytology, or histopathology. We combined recurrences and progression in the current study because the distinction between recurrences and progression was not always possible due to the study's retrospective nature. The location of recurrence was scored as follows: (1) local (penis only), (2) regional (inguinal or pelvic LNs), or (3) distant (any other location, including lymphangitis carcinomatosis). In patients with multiple recurrences, the most distant site was scored in the following order: distant, regional, and local. The location(s) of distant metastases, which could be multiple locations per patient, was recorded separately. Infield recurrences occurred in treated LN basins, whereas outfield recurrences occurred in untreated LN basins or distant sites.

Categorical variables were expressed as counts and percentages, and continuous variables were reported as the medians and interquartile ranges (IQRs). Unadjusted Cox regression was used to calculate hazard ratios (HRs) for the time to recurrence for different treatments and tumour characteristics. A competing risk analysis was used to evaluate the time to recurrence per location, and a Kaplan-Meier analysis was used to assess overall survival (OS) from the start of treatment and from the first recurrence. A comparison of survival curves was performed using the log-rank test. The recurrence pattern was analysed by taking the most distant recurrence within a patient and calculating the number as a crude percentage of the total number of patients with any recurrence (ie, conditional on developing a recurrence). Analyses were performed using R version 4.0.3 (R Foundation for Statistical Computing, Vienna, Austria).

## 3 Results

**Supplementary Figure 1:**
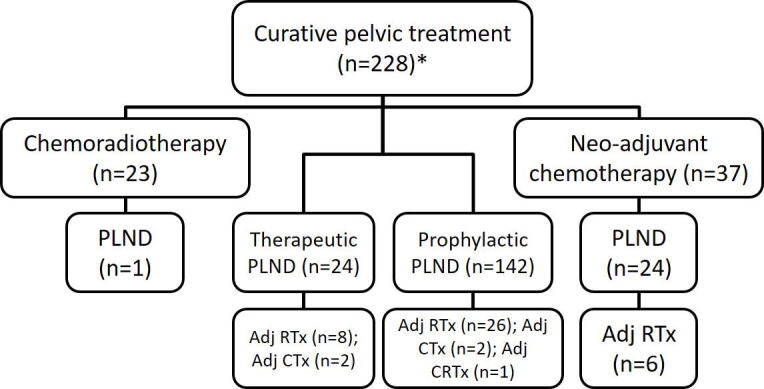
Flow diagram of treatments. *Two patients with neo-adjuvant radiotherapy, one followed by PLND, are not depicted in this diagram. PLND = pelvic lymph node dissection; Adj = adjuvant; RTx = Radiotherapy; CTx = chemotherapy; CRTx = chemoradiation.

In total, 228 patients were included. Of these patients, 154 (68%) underwent pelvic treatment as part of their first treatment (Table 1). Most patients (142; 62%) were treated with prophylactic PLND (Supplementary Fig. 1). The median follow-up of (surviving) patients was 79 (minimum 22; IQR 62–137) mo. The main treatment modalities in relation to the treatment period are provided in Supplementary Table 1.

**Table 1:** Baseline characteristics of 228 patients treated on their pelvis with curative intent

Age, median (IQR)	65 (57–71)
Treatment period, *n* (%)	
1969–1987	9 (3.9)
1988–1993	13 (5.7)
1994–2000	30 (13)
2001–2012	139 (61)
2013–2016	37 (16)
Pelvic treatment as part of first treatment, *n* (%)	154 (68)
Pathological T stage, *n* (%)	
pT1	29 (13)
pT2	100 (44)
pT3	75 (33)
pT4	10 (4.4)
pTx	14 (6.1)
Differentiation, *n* (%)	
Grade 1	34 (15)
Grade 2	102 (45)
Grade 3	77 (34)
Unknown	15 (6.6)
Positive primary tumour resection margin, *n* (%)	24 (11)
Clinical N stage, *n* (%)	
cN0	31 (14)
cN1	86 (38)
cN2	40 (18)
cN3	71 (31)
Suspicious pelvic nodes on imaging, *n* (%)	
None	47 (21)
Unilateral	45 (20)
Bilateral	20 (8.8)
≥2 tumour-positive inguinal lymph nodes per groin at pathology, *n* (%)	
Unilateral	94 (41)
Bilateral	44 (19)
Inguinal ENE at pathology, *n* (%)	
Unilateral	85 (37)
Bilateral	34 (15)

IQR = interquartile range; ENE = extranodal extension.

### 3.1 Predictors of recurrence

A recurrence was diagnosed in 138 (61%) patients during follow-up. The median recurrence-free survival after the start of pelvic treatment was 11 (7.7–18) mo. Within 2 yr, 97% of recurrences occurred. The risk of recurrence increased with pathological N stage, presence of positive pathological pelvic LNs, and the presence of ENE (Table 2). Subgroup analyses of only prophylactic PLNDs (*n* = 142) showed similar results to the entire cohort (Supplementary Table 2).

**Table 2:** Unadjusted relation between recurrence and patient, treatment, and tumour characteristics

	No recurrence	Recurrence	HR (95% CI)	*p* value
*n* (%)	90 (39)	138 (61)		
Age, median (IQR)	65 (59–73)	64 (55–70)	0.98 (0.96–1.0)	0.02
*Characteristics known before pelvic treatment*				
Treatment period, *n* (%)				
1969–1987	3 (33)	6 (67)	0.95 (0.37–2.5)	0.92
1988–1993	9 (69)	4 (31)	0.27 (0.08–0.91)	0.03
1994–2000	11 (37)	19 (63)	0.93 (0.51–1.7)	0.81
2001–2012	56 (40)	83 (60)	0.75 (0.48–1.2)	0.19
2013–2016	11 (30)	26 (70)	Ref	
Moment of treatment, *n* (%)				
First treatment	59 (38)	95 (62)	Ref	
Recurrence treatment	31 (42)	43 (58)	0.89 (0.62–1.3)	0.56
Main treatment modality, *n* (%)				
Chemoradiation	7 (30)	16 (70)	Ref	
Neoadjuvant chemotherapy	15 (41)	22 (59)	0.71 (0.37–1.4)	0.32
Prophylactic PLND	56 (39)	86 (61)	0.80 (0.47–1.4)	0.43
Therapeutic PLND	10 (42)	14 (58)	0.84 (0.41–1.7)	0.63
Suspicious pelvic nodes on imaging, *n* (%)				
None	19 (40)	28 (60)	Ref	
Unilateral	10 (22)	35 (78)	1.6 (0.97–2.6)	0.067
Bilateral	9 (45)	11 (55)	0.89 (0.43–1.8)	0.75
*Characteristics known after pelvic treatment*				
Adjuvant radiotherapy, *n* (%)				
No	71 (44)	92 (56)	Ref	
Yes	11 (27)	30 (73)	1.3 (0.86–2.0)	0.21
Differentiation, *n* (%)				
Good	15 (44)	19 (56)	Ref	
Intermediate	44 (43)	58 (57)	1.1 (0.62–1.8)	0.83
Poor	23 (30)	54 (70)	1.4 (0.80–2.4)	0.25
Pathological N stage, *n* (%)				
pN0	4 (100)	0 (0)		
pN1	13 (87)	2 (13)	0.10 (0.03–0.42)	0.002
pN2	18 (64)	10 (36)	0.28 (0.14–0.57)	<0.001
pN3	33 (27)	88 (73)	Ref	
pNx	22 (37)	38 (63)	0.72 (0.49–1.1)	0.11
Pathology pelvic nodes, *n* (%)				
Negative	66 (55)	54 (45)	Ref	
Positive	15 (21)	58 (79)	2.7 (1.8–3.9)	<0.001
ENE, *n* (%)				
Absent	51 (61)	32 (39)	Ref	
Present	35 (28)	88 (72)	2.6 (1.7–4.0)	<0.001

CI = confidence interval; HR = Hazard ratio; IQR = interquartile range; PLND = pelvic lymph node dissection; ENE = extranodal extension; Ref = reference.

### 3.2 Site of recurrence

**Fig. 1:**
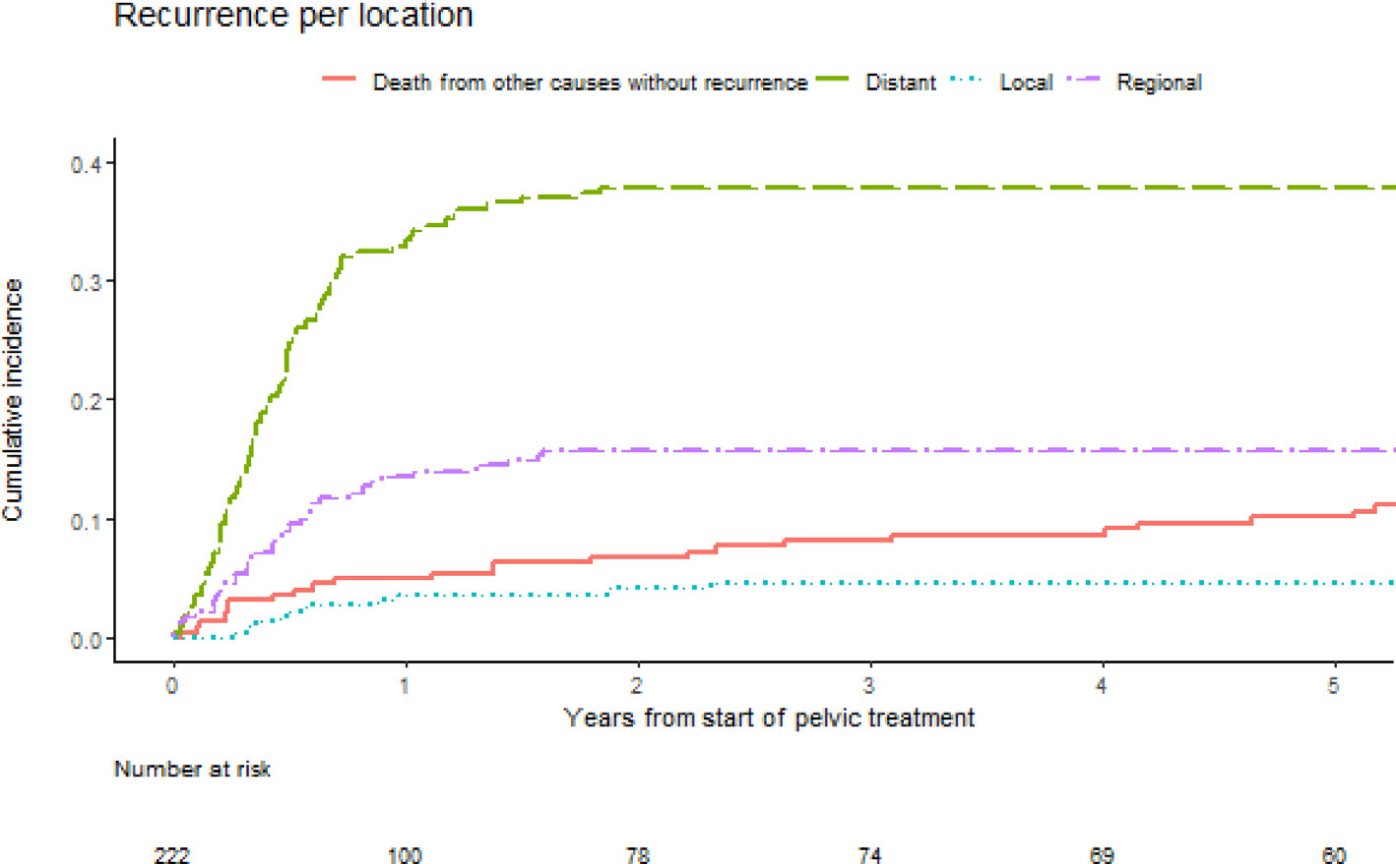
Cumulative incidence curves of the competing risk analyses for different recurrence locations after the start of pelvic treatment.

**Fig. 2:**
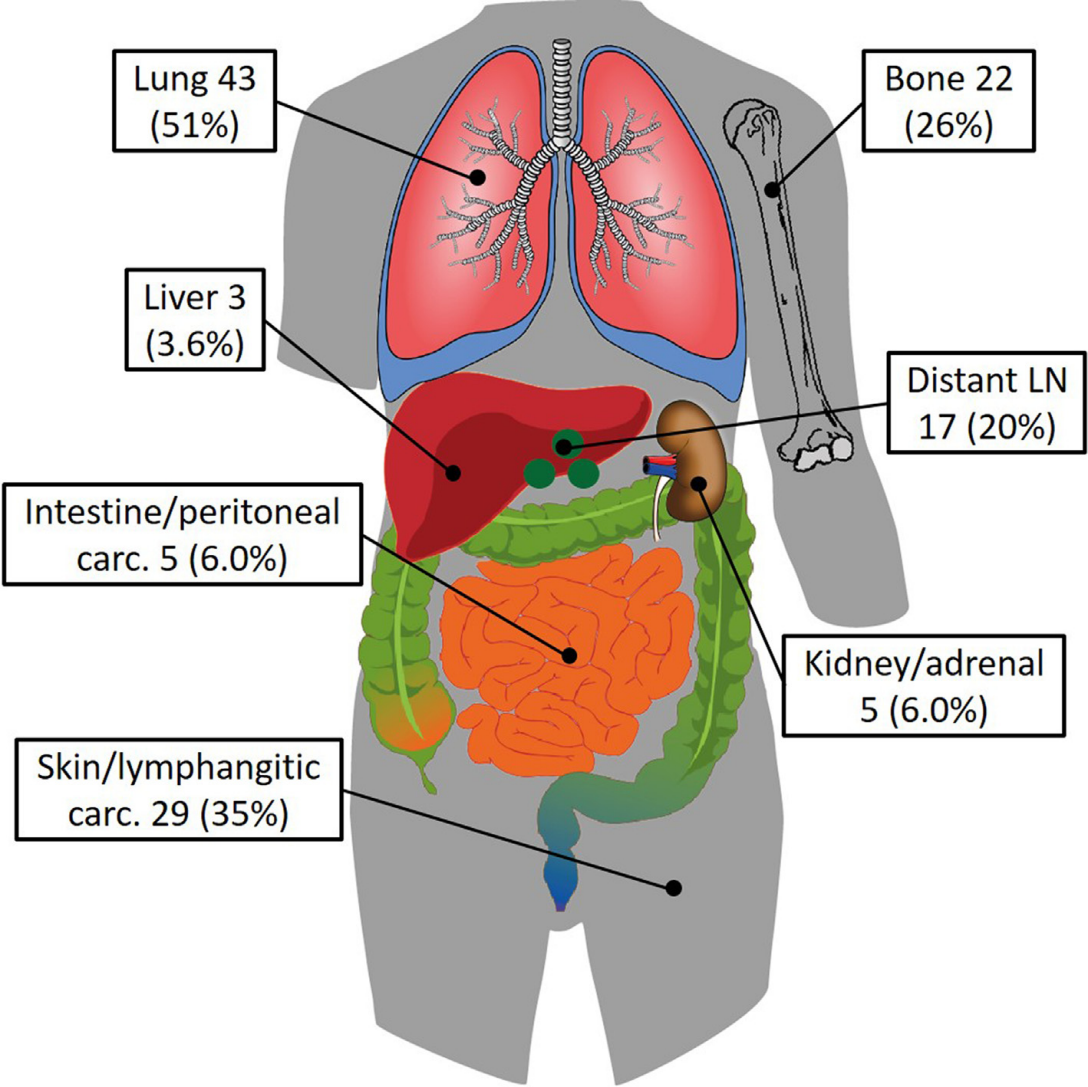
Locations of metastasis of 84 patients with a distant recurrence. Patients could have multiple distant locations. Percentages are the number of patients at a location divided by the total number of patients with distant metastasis. carc. = carcinomatosis; LN = lymph node.

In six patients, the location of recurrence was unknown (3%). One year after pelvic treatment, 5.0% of patients died from other causes without a recurrence, 4% had local recurrence, 14% had a regional recurrence, and 33% had a distant recurrence (Fig. 1). At the end of follow-up, the distribution of the most distant location of the first recurrence was as follows: local 12 (9%), regional 36 (27%), and distant 84 (64%). There was no statistically significant difference between the applied treatment modalities. Recurrences were distant more often in patients with ENE (67%) than in patients without ENE (45%). In patients with negative pathological pelvic nodes at therapeutic or prophylactic PLND, recurrences were distant in a majority of patients (57%). In patients with pathological positive pelvic nodes, this percentage of distant recurrences was only slightly higher (63%; Table 3). Subgroup analyses of prophylactic PLNDs showed similar results (Supplementary Table 3). The infield control rate of the 184 nonsystemically treated patients was 61% (113/184). Simultaneously, more than half of these patients (109/184, 59%) developed a recurrence, with 35% (38/109) being solitary and outside of the field of treatment (Supplementary Table 4). The lungs were the most frequently diagnosed distant metastasis site (43/84 [51%]; Fig. 2).

**Table 3:** Most distant location of the first recurrence after pelvic treatment

	Local	Regional	Distant
*n* (%)	12 (9.1)	36 (27)	84 (64)
Age, median (IQR)	65 (56–74)	67 (54–71)	63 (56–68)
*Characteristics known before pelvic treatment*			
Treatment period, *n* (%)			
1969–1987	0 (0)	3 (60)	2 (40)
1988–1993	0 (0)	0 (0)	3 (100)
1994–2000	1 (5.3)	5 (26)	13 (68)
2001–2012	11 (14)	25 (32)	43 (54)
2013–2016	0 (0)	3 (12)	23 (88)
Moment of treatment, *n* (%)			
Primary treatment	7 (7.5)	25 (27)	61 (66)
Recurrence treatment	5 (13)	11 (28)	23 (59)
Main treatment modality, *n* (%)			
Chemoradiotherapy	0 (0)	2 (13)	14 (88)
Neoadjuvant chemotherapy	0 (0)	6 (32)	13 (68)
Prophylactic PLND	11 (13)	24 (29)	48 (58)
Therapeutic PLND	1 (7.1)	4 (29)	9 (64)
Suspicious pelvic nodes on imaging, *n* (%)			
None	3 (11)	7 (25)	18 (64)
Unilateral	1 (3)	8 (24)	25 (74)
Bilateral	0 (0)	2 (20)	8 (80)
*Characteristics known after pelvic treatment*			
Adjuvant radiotherapy, *n* (%)			
No	11 (13)	28 (32)	49 (56)
Yes	1 (3.6)	6 (21)	21 (75)
Differentiation, *n* (%)			
Good	1 (5.9)	10 (59)	6 (35)
Intermediate	4 (7.1)	12 (21)	40 (71)
Poor	7 (13)	12 (23)	33 (63)
Pathological N stage, *n* (%)			
pN1	0 (0)	1 (50)	1 (50)
pN2	4 (44)	3 (33)	2 (22)
pN3	8 (9.0)	24 (28)	54 (63)
pNx	0 (0)	8 (23)	27 (77)
Pathology pelvic nodes, *n* (%)			
Negative	9 (17)	14 (26)	30 (57)
Positive	3 (5.4)	18 (32)	35 (63)
ENE, *n* (%)			
Absent	6 (19)	11 (35)	14 (45)
Present	6 (7.0)	22 (26)	58 (67)

IQR = interquartile range; ENE = extranodal extension; PLND = pelvic lymph node dissection.

### 3.3 Survival

**Fig. 3:**
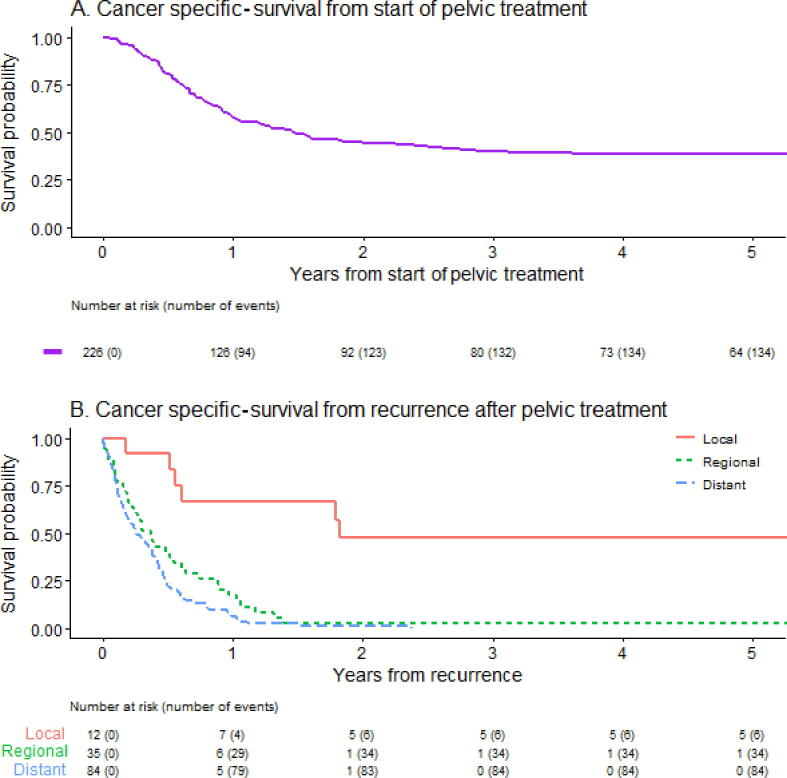
Cancer-specific survival from (A) the start of pelvic treatment and (B) the first recurrence after pelvic treatment.

**Supplementary Figure 2:**
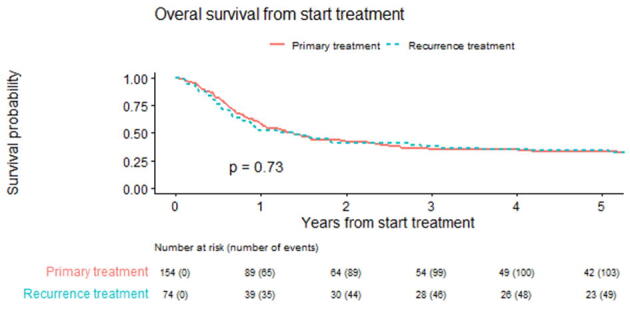
Overall survival from the start of treatment for patients undergoing pelvic treatment as primary treatment or as part of the treatment of a recurrence.

The cause of death was missing for two patients. The median OS and CSS from the start of pelvic treatment for all patients were 17 (95% confidence interval [CI] 12–22) and 18 (95% CI 13–29) mo, respectively. The 5-yr OS and CSS were 33% (95% CI 28–40%) and 39% (95% CI 33–46%; Fig. 3A), respectively. Patients for whom pelvic treatment was part of their primary treatment had similar median OS to patients for whom pelvic treatment was part of treatment for a pelvic recurrence after prior local and inguinal therapy (9.3 vs 14 mo; *p* = 0.56; Supplementary Fig. 2). After pelvic treatment, all but one patient with a regional or distant recurrence died of PeCa. The median CSS after recurrence by recurrence location was 22 (95% CI 7.4–infinite), 4.4 (95% CI 2.7–7.7), and 3.1 (95% CI 2.1–4.8) mo for local, regional, and distant recurrences, respectively (Fig. 3B).

## 4 Discussion

This study evaluates the disease course after pelvic treatment for locally advanced PeCa. More than half of patients who undergo pelvic treatment develop a recurrence despite extensive (multimodal) treatment, of which more than half recurrences are at distant sites.

The recurrence rate after pelvic treatment (61%) in this study is, as expected with more advanced disease, much higher than after inguinal LN dissection (ILND; 31%), as reported by Chakiryan et al [17]. Likewise, the number of distant recurrences as a fraction of the total number of recurrences is higher after pelvic treatment (64%) than after ILND (46%) [8]. The time to recurrence was also shorter after pelvic treatment (97% of recurrences within 24 mo) than after ILND (95% of recurrences within 48 mo) [17].

The different treatment modalities applied throughout the years did not show different hazard rates for recurrence. We acknowledge that this retrospective study suffers from inherent biases due to developing treatments and diagnostics over time. Nevertheless, it is intriguing from a clinical perspective that there was no statistically significant difference in OS between patients who received primary pelvic treatment or patients treated for a recurrence requiring pelvic treatment. Similar survival might be caused by a selection bias as the patients who have a recurrence and receive curative pelvic treatment have not yet developed distant metastasis and might thus have a less aggressive tumour.

The strong tendency for distant spread is even more intriguing from a biological perspective. Distant recurrence occurred in 26/94 (28%) patients without pathological pelvic nodal involvement at prophylactic or therapeutic pelvic node dissection, which is more than half of all recurrences in this group. There are no distant metastases without regional LN metastasis, as patients had either inguinal metastasis prior to pelvic treatment or regional LN metastasis synchronous with the distant metastasis. These findings show that the efficacy of surgery alone as pelvic treatment is limited and stress the urgent need for more effective (systemic) treatment options. From our analysis, it remains unclear which factors are responsible for this biological behaviour.

After the diagnosis of a recurrence, only a minority of patients survived longer than 1 yr. Patients with a regional recurrence did slightly better than those with distant recurrences. Surprisingly, over half of the patients with only a local recurrence at first also died within 2 yr after the start of pelvic treatment, suggesting a lack of regional and systemic disease control after pelvic treatment.

This study is limited by its retrospective nature and the inherent missing data and selection bias accompanying this type of research. The variation in diagnostic imaging and treatment protocols within the almost 50-yr period, as described in the Patients and methods section, further limits this study. Owing to limited patient numbers in some groups, recurrence patterns between treatment modalities could not be compared directly. Nevertheless, our study still represents one of the largest cohorts of PeCa patients who underwent pelvic treatment and underlined the need for new, more effective systemic treatment options for patients with an indication for pelvic treatment.

## 5 Conclusions

This study highlights a strong tendency for recurrence and systemic spread in PeCa patients who underwent pelvic treatment. Despite treatment, these patients' prognosis remains poor, emphasising the need for more effective systemic treatment strategies.

  ***Author contributions*:** Oscar R. Brouwer had full access to all the data in the study and takes responsibility for the integrity of the data and the accuracy of the data analysis.

*Study concept and design*: de Vries, Ottenhof, Horenblas.

*Acquisition of data*: de Vries, Ottenhof.

*Analysis and interpretation of data*: de Vries, Horenblas, Brouwer.

*Drafting of the manuscript*: de Vries.

*Critical revision of the manuscript for important intellectual content*: Brouwer, Horenblas, Rafael, Pos, van Rhijn, Moonen, Graafland, de Feijter, Schaake, Ottenhof.

*Statistical analysis*: van Werkhoven.

*Obtaining funding*: Horenblas, Brouwer.

*Administrative, technical, or material support*: Rafael.

*Supervision*: Horenblas, Brouwer.

*Other*: None.

  ***Financial disclosures:*** Oscar R. Brouwer certifies that all conflicts of interest, including specific financial interests and relationships and affiliations relevant to the subject matter or materials discussed in the manuscript (eg, employment/affiliation, grants or funding, consultancies, honoraria, stock ownership or options, expert testimony, royalties, or patents filed, received, or pending), are the following: None.

  ***Funding/Support and role of the sponsor*:** None.

  ***Acknowledgments*:** We thank Tony van de Velde for database management.
