# Supplementary material for: Patterns of Recurrence and Survival After Pelvic Treatment for Locally Advanced Penile Cancer
Source: Eur Urol Open Sci. 2022 Dec 15;47:29–35. doi: 10.1016/j.euros.2022.11.005 (PMC9806705; doi:10.1016/j.euros.2022.11.005)
Supplement: Supplementary Tables [file mmc1.docx]

| **Supplementary Table 1. Treatment modality per treatment period of 228 patients** | | | | | | |  |
| --- | --- | --- | --- | --- | --- | --- | --- |
|  |  | Treatment period | | | | |  |
|  | Total | 1969-1987 | 1988-1993 | 1994-2000 | 2001-2012 | 2013-2016 | |
| Total, n | 228 | 9 | 13 | 30 | 139 | 37 |  |
| Main treatment modality, n |  |  |  |  |  |  |  |
| Chemoradiation | 23 | 0 | 0 | 0 | 0 | 23 |  |
| Neo-adjuvant chemotherapy | 37 | 1 | 0 | 3 | 33 | 0 |  |
| Neo-adjuvant radiotherapy | 2 | 1 | 1 | 0 | 0 | 0 |  |
| Prophylactic PLND | 142 | 7 | 11 | 21 | 92 | 11 |  |
| Therapeutic PLND | 24 | 0 | 1 | 6 | 14 | 3 |  |
| PLND = pelvic lymph node dissection | | | | | | |  |

| **Supplementary Table 2. Unadjusted relation between recurrence and patient, treatment and tumour characteristics in patients who had a prophylactic PLND** | | | | |
| --- | --- | --- | --- | --- |
|  |  |  |  |  |
|  | No recurrence | Recurrence | HR (95% CI) | p-value |
| n (%) | 56 (39) | 86 (61) |  |  |
| Age, median (IQR) | 66 (61-73) | 64 (54-70) | 0.98 (0.96-1.0) | 0.032 |
|  |  |  |  |  |
| *Characteristics known before pelvic treatment* | |  |  |  |
| Treatment period, n (%) |  |  |  |  |
| 1969-1987 | 1 (14) | 6 (86) | 1.3 (0.42-4.2) | 0.62 |
| 1988-1993 | 7 (64) | 4 (36) | 0.32 (0.08-1.3) | 0.10 |
| 1994-2000 | 7 (33) | 14 (67) | 0.98 (0.39-2.4) | 0.96 |
| 2001-2012 | 37 (40) | 55 (60) | 0.81 (0.37-1.8) | 0.60 |
| 2013-2016 | 4 (36) | 7 (64) | ref |  |
| Moment of treatment, n (%) |  |  |  |  |
| First treatment | 35 (38) | 57 (62) | ref |  |
| Recurrence treatment | 21 (42) | 29 (58) | 0.80 (0.51-1.3) | 0.35 |
|  |  |  |  |  |
| *Characteristics known after pelvic treatment* | |  |  |  |
| Adjuvant radiotherapy, n (%) |  |  |  |  |
| No | 49 (43) | 66 (57) | ref |  |
| Yes | 7 (26) | 20 (74) | 1.4 (0.81-2.3) | 0.25 |
| Differentiation, n (%) |  |  |  |  |
| Good | 9 (39) | 14 (61) | ref |  |
| Intermediate | 30 (42) | 42 (58) | 1.0 (0.55-1.9) | 0.95 |
| Poor | 13 (33) | 27 (68) | 1.3 (0.65-2.5) | 0.50 |
| Pathological N-stage, n (%) |  |  |  |  |
| pN1 | 12 (86) | 2 (14) | 0.12 (0.03-0.50) | 0.003 |
| pN2 | 16 (62) | 10 (38) | 0.33 (0.17-0.66) | 0.002 |
| pN3 | 28 (27) | 74 (73) | ref |  |
| Pathology pelvic nodes, n (%) |  |  |  |  |
| negative | 47 (50) | 47 (50) | ref |  |
| positive | 9 (19) | 39 (81) | 2.5 (1.6-3.9) | < 0.001 |
| ENE, n (%) |  |  |  |  |
| absent | 31 (61) | 20 (39) | ref |  |
| present | 25 (27) | 66 (73) | 2.7 (1.6-4.5) | < 0.001 |
| HR = Hazard ratio; CI = confidence interval; IQR = interquartile range; ENE = extranodal extension | | | | |

| **Supplementary Table 3. Most distant location of the first recurrences after pelvic treatment in patients who had a prophylactic PLND** | | | |
| --- | --- | --- | --- |
|  | Local | Regional | Distant |
| n (%) | 11 (13) | 24 (29) | 48 (58) |
| Age, median (IQR) | 62 (56-75) | 69 (50-71) | 64 (55-68) |
|  |  |  |  |
| *Characteristics known before pelvic treatment* | |  |  |
| Treatment period, n (%) |  |  |  |
| 1969-1987 | 0 (0) | 3 (60) | 2 (40) |
| 1988-1993 | 0 (0) | 0 (0) | 3 (100) |
| 1994-2000 | 1 (7.1) | 4 (29) | 9 (64) |
| 2001-2012 | 10 (19) | 16 (30) | 28 (52) |
| 2013-2016 | 0 (0) | 1 (14) | 6 (86) |
| Moment of treatment, n (%) |  |  |  |
| First treatment | 6 (11) | 17 (30) | 34 (60) |
| Recurrence treatment | 5 (19) | 7 (27) | 14 (54) |
|  |  |  |  |
| *Characteristics known after pelvic treatment* | |  |  |
| Adjuvant radiotherapy, n (%) |  |  |  |
| No | 10 (15) | 19 (29) | 36 (55) |
| Yes | 1 (5.6) | 5 (28) | 12 (67) |
| Differentiation, n (%) |  |  |  |
| Good | 1 (7.7) | 7 (54) | 5 (38) |
| Intermediate | 4 (9.8) | 8 (20) | 29 (71) |
| Poor | 6 (23) | 8 (31) | 12 (46) |
| Pathological N-stage, n (%) |  |  |  |
| pN1 | 0 (0) | 1 (50) | 1 (50) |
| pN2 | 4 (44) | 3 (33) | 2 (22) |
| pN3 | 7 (9.7) | 20 (28) | 45 (63) |
| Pathology pelvic nodes, n (%) |  |  |  |
| negative | 8 (18) | 12 (26) | 26 (57) |
| positive | 3 (8.1) | 12 (32) | 22 (59) |
| ENE, n (%) |  |  |  |
| absent | 6 (32) | 7 (37) | 6 (32) |
| present | 5 (7.8) | 17 (27) | 42 (66) |
| IQR = interquartile range; ENE = extranodal extension | | | |

| **Supplementary Table 4. In or outfield recurrence of 184 not systemically treated patients** | | | | |
| --- | --- | --- | --- | --- |
|  | No recurrence | Solitary infield | Solitary outfield | Both |
| Surgery, n (%) | 57 (45) | 25 (20) | 24 (19) | 20 (16) |
| Chemoradiation, n (%) | 6 (26) | 4 (17) | 5 (22) | 8 (35) |
| Surgery and (Chemo-) radiotherapy, n (%) | 12 (34) | 3 (8.6) | 9 (26) | 11 (31) |
